# Single-cell RNA datasets and bulk RNA datasets analysis demonstrated *C1Q*+ tumor-associated macrophage as a major and antitumor immune cell population in osteosarcoma

**DOI:** 10.3389/fimmu.2023.911368

**Published:** 2023-02-06

**Authors:** Jihao Tu, Duo Wang, XiaoTian Zheng, Bin Liu

**Affiliations:** Department of Hand and Foot Surgery, The First Hospital of Jilin University, Changchun, Jilin, China

**Keywords:** tumor-associated macrophages, osteosarcoma, immune infiltration, biomarker, single-cell sequencing technology

## Abstract

**Background:**

Osteosarcoma is the most frequent primary bone tumor with a poor prognosis. Immune infiltration proved to have a strong impact on prognosis. We analyzed single-cell datasets and bulk datasets to confirm the main immune cell populations and their properties in osteosarcoma.

**Methods:**

The examples in bulk datasets GSE21257 and GSE32981 from the Gene Expression Omnibus database were divided into two immune infiltration level groups, and 34 differentially expressed genes were spotted. Then, we located these genes among nine major cell clusters and their subclusters identified from 99,668 individual cells in single-cell dataset GSE152048 including 11 osteosarcoma patients. Especially, the markers of all kinds of myeloid cells identified in single-cell dataset GSE152048 were set to gene ontology enrichment. We clustered the osteosarcoma samples in the TARGET-OS from the Therapeutically Applicable Research to Generate Effective Treatments dataset into two groups by complete component 1q positive macrophage markers and compared their survival.

**Results:**

Compared with the low-immune infiltrated group, the high-immune infiltrated group showed a better prognosis. Almost all the 34 differentially expressed genes expressed higher or exclusively among myeloid cells. A group of complete component 1q-positive macrophages was identified from the myeloid cells. In the bulk dataset TARGET-OS, these markers and the infiltration of complete component 1q-positive macrophages related to longer survival.

**Conclusions:**

Complete component 1q-positive tumor-associated macrophages were the major immune cell population in osteosarcoma, which contributed to a better prognosis.

## 1 Introduction

Osteosarcoma (OS) represents the most frequent and primary bone sarcoma, which primarily affects children, adolescents, and young adults (1). The standard therapy for OS, comprising surgery and chemotherapy, was established in the 1980s and resulted in long-term survival in >60% of patients presenting with localized disease (2); however, limited therapeutic progress has been made since that time.

Infiltrating immune and stromal cells are essential for OS progression (1). The immune infiltration level was considered an important factor in response to immunotherapy and prognosis. Analyses of the tumor microenvironment (TME) of OS consistently demonstrate an immune cell infiltration consisting of both macrophages and T cells (1, 3, 4). Primary OS is demonstrated as “immune deserts,” devoid of T cells and NK cells (5, 6). Instead, myeloid cells were observed in large quantities (7). In the OS microenvironment, tumor-associated macrophages (TAMs) play a critical role in immunoreaction (8). However, among contradictory conclusions, it is still not clear if these myeloid cells or the TAMs contribute to tumor growth or tumor limitation.

In the analysis of the public dataset GSE150248, we found that TAMs were an essential population in the TME of OS. Generally, macrophages are considered as a plastic cell type because they can be polarized into different phenotypes. M1-type macrophages (M1) and M2-type macrophages (M2) are two major kinds of them. M1 can be induced by pathogen‐associated patterns such as lipopolysaccharides and interferon‐γ. M1 highly expresses interleukin 6 (*IL‐6*), *IL‐1β*, and tumor necrosis factor, which facilitate a proinflammatory response. M2 can be induced by *IL‐4* and *IL‐13*, which turn on the expression of anti‐inflammatory cytokines, such as *IL‐10* and *ARG1*. These are considered immune suppression and pro-tumor signals (9). Reprograming M2-like TAMs to M1-like TAMs exerts a synergistic effect in radiotherapy and overcoming chemoresistance in breast cancer (10–12). Macrophages can also be divided by where they were produced. TAMs are proved to be of dichotomous origin, from *in situ* proliferation marked by *FOLR2* and the differentiation of circulating monocytes marked by *TREM2* (13–15). It is reported that the M1 or M2 paradigm is an oversimplification, Tissue-resident macrophages are far more complex cells with a full range of identities and activation states (16). In human breast cancer, tissue-resident *FOLR2*+ macrophages instead of M1 or M2 are proved to associate with *CD8*+ T-cell infiltration and better prognosis (17).

Complete component 1q (*C1Q*), one of the three first components of the classical pathway, modulates both inflammation and repair progress (18). *C1Q* is a marker of a particular subpopulation of tissue-resident macrophages and TAMs, which often expresses *CD206*, *HLA-DR*, *SEPP1*, *FOLR2*, and *APOE* (19). In cancer, *C1Q* is usually regarded as a cancer-promoting factor (15, 20). In the classical pathway, *C1Q* generates the C5a production, which was proved as an immunosuppression and angiogenesis factor in cancer progression (21–23). *C1Q* can function as a pattern recognition receptor to apoptotic cells and extracellular vesicles before a non-inflammatory clearance by macrophages. In this case, macrophages produce M2 markers such as *IL-10* and *TGFβ* (24).

Here, we explored the immune-related genes of OS. Especially, we mapped these genes among all the cell populations through a combination of bulk-sequencing and single-cell sequencing technology. We found that *C1Q*+ TAMs are the main immune cells in the OS TME. In detail, *C1Q* is an obvious immune-related gene expressed exclusively by myeloid cells. Moreover, this research found that *C1Q*, different from its pro-tumor characteristic in other cancers (15), seems to be an antitumor factor in OS. *C1Q*+ TAMs promote *CD8+* T-cell dysfunction and tumor growth in the Lewis lung carcinoma mouse model (25). However, we noticed that *C1Q*+ TAMs act as an antitumor cell population in OS.

## 2 Materials and methods

### 2.1 Datasets for analysis and derivation of the gene list

**Figure 1:**
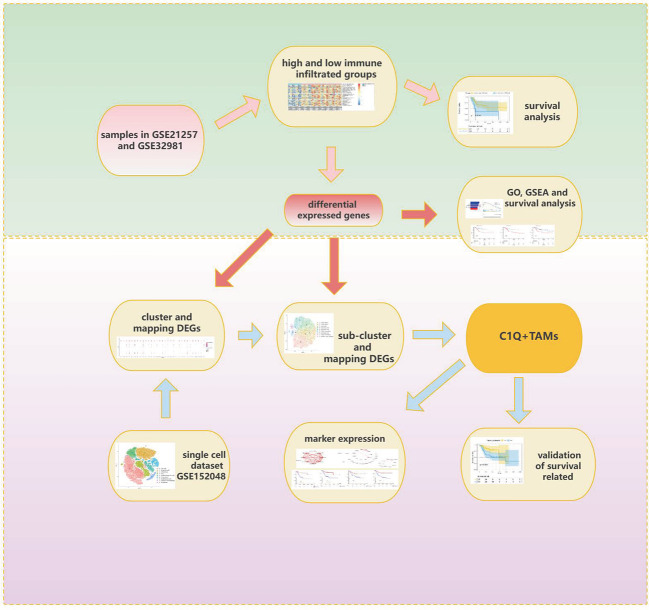
Workflow of this research.

Clinical and transcriptome data of OS patients were downloaded from the Therapeutically Applicable Research to Generate Effective Treatments (TARGET) database (https://ocg.cancer.gov/programs/target) and the Gene Expression Omnibus (GEO) database (https://www.ncbi.nlm.nih.gov/geo/). Dataset TARGET-OS contains 88 samples with both complete survival information and expression profiles. Dataset GSE21257 contains 53 OS samples with survival information and expression profiles. Dataset GSE32981 contains 23 samples with only expression profiles. Specific clinical information of 88 samples in the TARGET database and 53 samples in the GSE21257 dataset is separately listed in **Supplementary Tables S7, S8**. Dataset GSE152048, a single-cell dataset, contains tumor samples from 11 OS patients (five men and six women, 11–38 years old). There are eight osteoblastic OS lesions, including six primary, one recurrent, and one lung metastatic lesions, and three chondroblastic OS lesions including one primary, one recurrent, and one lung metastasis site. The workflow of this research is provided in **Figure 1**.

### 2.2 Samples clustered into high- and low-immune infiltrated groups and their immune cell scores evaluated

The samples in datasets GSE21257 and GSE32981 were clustered into high- and low-immune infiltrated groups by R. We used identified immune metagenes (26) and function hclust(x, method = “complete”) and cutree(x, k = 2) to divide the samples into two groups. Then, the overall survival was compared between two groups by the R package survival (https://CRAN.R-project.org/package=survival) and survminer (https://CRAN.R-project.org/package=survminer). The grouping of samples in dataset TARGET-OS was almost the same, except that the immune metagenes were replaced by *C1Q*+ TAM markers with a fold change larger than 0.25. Based on the normalized expression matrix, immune scores across OS specimens from the GSE21257 dataset were estimated using single-sample gene set enrichment analysis (ssGSEA). This algorithm infers the overall infiltration levels of immune cells in tumor tissues using gene expression signatures. The Kaplan–Meier overall survival curves were examined between groups, and the prognosis was compared by log-rank test.

### 2.3 Differential expression analysis, functional enrichment analysis, and gene set enrichment analysis

The limma, edgeR, and DESeq2 packages were applied for differential expression analysis (27–29). |Fold change (FC)| > 1.5 and adjusted p < 0.05 were set as the criteria of differentially expressed gene (DEG) identification. The enrichment analysis of DEGs was carried out *via* the clusterProfiler package, including Gene Ontology (GO) (30). Terms with adjusted p < 0.05 were significantly enriched. Gene set enrichment analysis (GSEA) evaluates microarray data at the level of gene sets. The DEGs identified from GSE21257 were used as the gene sets (30).

### 2.4 Single-cell data processing

Single-cell dataset GSE152048 was processed with the Seurat package (version 4.1.0; http://satijalab.org/seurat/). Each of the 11 samples generated a Seurat object by function Read10×. Next, we filtered out the cells with less than 300 expressed genes or with mitochondrial gene expression accounting for more than 10% of total expressed genes. The top 3,000 highly variable genes were picked up for the principal component analysis. Further, the doublets in each Seurat object were cleared out by the DoubletFinder package (version 2.0.3) (31). We integrated the 11 Seurat objects into one combined Seurat object by base function merge(). The batch effects were removed by the Harmony package (version 1.0). Functions FindNeighbors(x, reduction = “harmony”), FindClusters(x, resolution = 0.1), and FindAllMarkers(x) were applied to the cell clustering and cluster annotation. Fold change (FC) > 0.25 and adjusted p < 0.05 were set as the criteria of DEGs or markers between cell groups. 2D maps of the identified clusters were generated with the distributed Stochastic Neighbor Embedding or Uniform Manifold Approximation and Projection method. A similar procedure was applied during the subclustering analysis.

### 2.5 Cell–cell communication analysis with CellPhoneDB 2

CellPhoneDB 2 is a repository of ligand–receptor complexes and a statistical tool to predict the cell-type specificity of cell–cell communication *via* molecular interactions (32). The repository includes subunit architecture for both ligands and receptors, to accurately represent heteromeric complexes. We used CellPhoneDB 2 to calculate the interaction pairs between *C1Q*+ TAMs and the rest of the 13 clusters that gained after clustering and subclustering. Interaction pairs with a p-value less than 0.05 returned by CellPhoneDB 2 were picked up to draw an interaction bubble plot.

CellPhoneDB 2 was used in the python 3.7 environment; the rest of the analysis was presented using R version 4.1.2 (http://www.R-project.org) and its appropriate packages.

## 3 Results

### 3.1 High-immune infiltrated group showed a better prognosis in osteosarcoma

**Figure 2:**
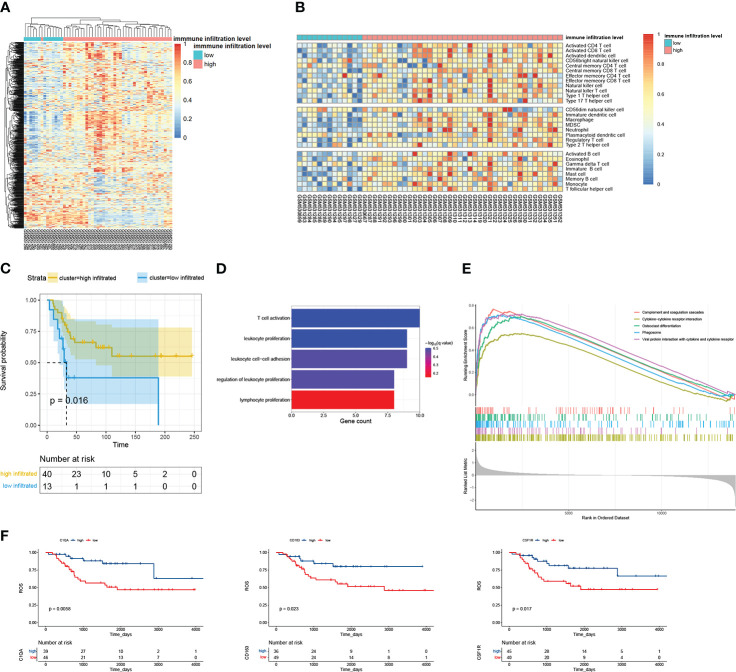
The overview of analyzing GSE21257. **(A)** There were 53 examples clustered into two groups by immune metagenes. **(B)** The score of 28 kinds of immune cells calculated by ssGSEA. The 53 examples were ordered by their immune groups instead of getting clustered. **(C)** The survival plot of high- and low- immune infiltrated groups. **(D)** Biological process enrichment of the 34 overlapped DEGs. **(E)** GSEA of the 34 overlapped genes; the top 5 terms were selected. All p-values are 1e-10, and all p.adjust are 1.447826e-09. **(F)** The survival plot of overlapped genes.

After clustering, 40 and 13 samples of dataset GSE21257 were divided into high- and low-immune infiltrated groups, respectively (**Figure 2A**). Then, we calculated the immune infiltration scores of these two groups by ssGSEA. The high-immune infiltrated group indeed showed a higher immune cell score, but it is kind of strange that all kinds of immune cell scores were lower in the low-immune infiltrated group (**Figure 2B**); especially, the low-immune infiltrated group indeed showed some highly expressed genes. Maybe it was one kind of immune cell that caused the highly expressed genes in both two groups. The high-immune infiltrated group had a better prognosis (**Figure 2C**). We performed a similar analysis progress, dividing samples and then calculating the immune scores using ssGSEA, on GSE32981 (**Figure S1**). There were 20 and 3 samples in GSE32981 that were divided into high- and low- immune infiltrated groups (**Figure S1A**). The high-immune infiltration group also showed a higher immune score (**Figure S1B**). A step further, DEGs between high-and low-immune infiltrated groups were respectively collected in datasets GSE21257 and GSE32981. The high-immune infiltrated group of GSE21257 got 146 higher expressed genes and 261 lower expressed genes. As for GSE32981, it got 110 higher expressed genes and 14 lower expressed genes. In a total of 240 higher expressed genes, 34 overlapped genes were picked up. We found no intersections between 275 lower expressed genes. Moreover, just like the survival plot depending on immune-related grouping, almost all of these 34 genes were bound up with better overall survival (**Figures 2F**, **S2**). GO biological process enrichment showed that those 34 gene genes were involved in the process of leukocyte and T-cell proliferation (**Figure 2D**). Further GSEA of DEG analyses showed the hallmarks of complement signaling, cytokine−cytokine receptor interaction, osteoclast differentiation, phagocytosis, and viral protein interaction with cytokine and cytokine receptor were highly enriched (**Figure 2E**).

### 3.2 Macrophages are the main immune cell population in osteosarcoma

**Figure 3:**
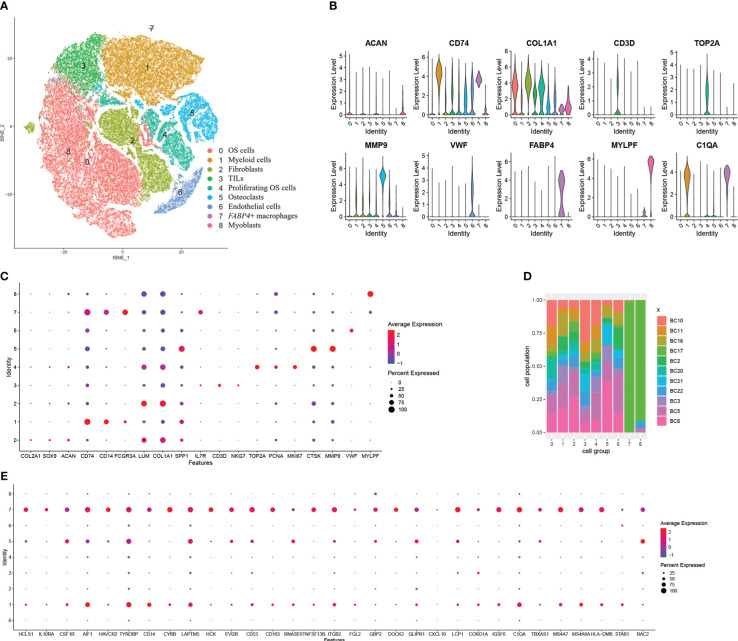
Single-cell transcriptomic analysis reveals the transcriptome of cells in the microenvironment of OS. **(A)** The t-distributed stochastic neighbor embedding (t-SNE) plot of the nine identified main cell types in OS lesions. **(B)** Violin plots showed the normalized expression levels of eight representative canonical markers across the nine clusters. **(C)** Representative marker expression in nine clusters of cells. Dot size indicates the proportion of cells expressing markers. Dot color shows the average expression level of the markers. **(D)** Distribution of the nine clusters among 11 patients with OS. **(E)** Dot plot of the 34 overlapped DEGs showing their expressing proportion and level among nine clusters.

To explore the source of higher expressed genes, we analyzed the cell population of OS with the single-cell sequencing dataset GSE152048. After initial quality control assessment and doublet removal, we obtained single-cell transcriptomes from a total of 99,668 cells. These cells were clustered into nine groups. They are as follows: (0) 37,939 OS cells highly express *SPP1*, *COL2A1*, *SOX9*, and *ACAN*; (1) 21,067 myeloid cells highly express *CD74*, *CD14*, and *FCGR3A*; (2) 13,667 fibroblasts highly express *COL1A1* and *LUM*; (3) 8,089 TILs including T and NK cells highly express *IL7R*, *CD3D*, and *NKG7*; (4) 7,699 proliferating OS cells highly express *TOP2A*, *PCNA*, and *MKI67*; (5) 7,307 osteoclasts highly express *MMP9* and *CTSK*; (6) 3,646 endothelial cells highly express *vWF*, (7) 129 *FABP4*+ macrophages highly express *FCGR3A* and *FABP4*; and (8) 125 myoblasts highly express MYPL (**Figures 3A–D**). The violin plots show the expression level of one representative marker gene of each cell group, sequentially, except *C1QA*. It is more convenient to compare the expression of markers by dot plot (**Figures 3B, C**). *COL1A1*, a marker of fibroblasts and OS cells, is mainly expressed in fibroblasts and also in OS cells. In addition, *ACAN* is mainly expressed in OS cells but also in fibroblasts. First, seven clusters are distributed evenly among 11 patients (**Figure 3D**). The *FABP4*+ macrophage group and myoblast group, with very little cell number, consist mainly of cells in sample BC17. It is worth noting that the *FABP4*+ macrophages have an unusually high number of detected genes. We also calculated the DEGs in each cell group and the GO terms these genes enriched (**Table S1**). The markers in myeloid cells were enriched in immune response, myeloid cell activation, and myeloid cell differentiation (**Table S2**). We tried to match the 34 higher expressed DEGs to the certain cell groups identified here. The dot plot showed that these genes were largely expressed by myeloid cells and *FABP4*+ macrophages (**Figure 3E**).

### 3.3 *C1QA* and other overlapped DEGs were mainly expressed by *C1Q*+ TAMs

**Figure 4:**
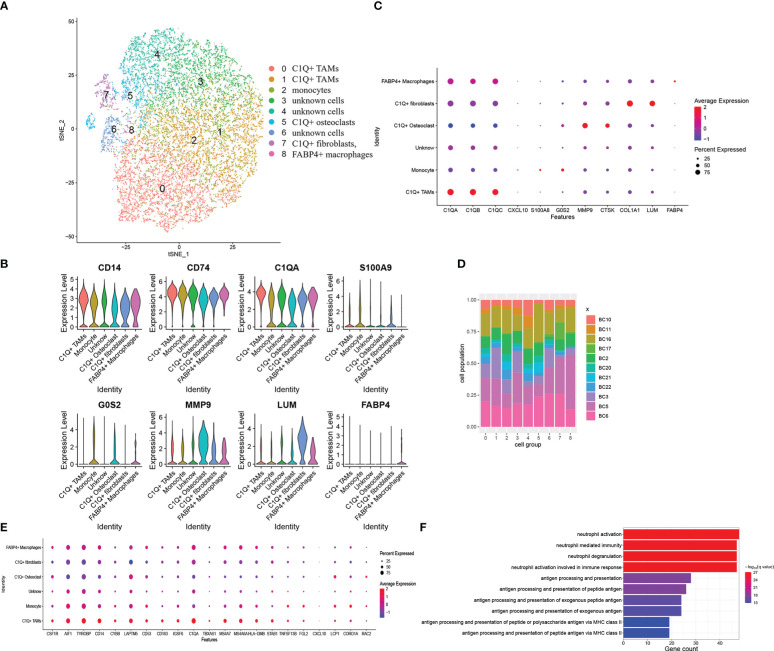
Myeloid cells subclustered and DEGs combined with these six cell groups. **(A)** The t-SNE plot of the nine identified clusters or six cell groups. **(B)** Violin plots showed the normalized expression levels of representative markers across the six cell groups **(C)** Representative markers expression in six cell groups. Dot size and color delivered the same meaning as the previous dot plot **(D)** Distribution of the nine clusters among 11 patients with OS. **(E)** Dot plot of the overlapped DEGs, which represented as C1Q+ TAM markers. The plot showed expressing proportion and level among six cell groups of these DEGs. **(F)** Biological process enrichment of *C1Q*+ TAM markers.

As DEGs were expressed by myeloid cells and *FABP4*+ macrophages, we took the myeloid cell group to a subcluster analysis similar to the previous step. Myeloid cells were divided into nine clusters when the resolution was set as 0.5. We identified six cell groups from the nine clusters (**Figures 4A–D**). They are (1) 9,601 C1Q+ TAMs with high *C1Q* expression in clusters 0 and 1; (2) 3,516 monocytes with low *C1Q* expression and high *G0S2* and *S100A9* expression in cluster 2; (3) 1,072 *C1Q*+ osteoclasts with high *C1Q*, *MMP9*, and *CTSK* expression in cluster 5; (4) 609 *C1Q*+ fibroblasts with high *C1Q*, *COL1A1*, and *LUM* expression in cluster 7; (5) 79 *FABP4*+ macrophages with high FABP4 expression in cluster 8; and (6) 7,418 unknown cells in clusters 3, 4, and 6 (**Figures 4A–D**). The violin plot shows that *CD14*, *CD74*, and *C1Q* are expressed in all groups (**Figure 4B**). *C1Q* expresses the highest in *C1Q*+ TAMs and lower in monocytes and *C1Q*+ osteoclasts (**Figure 4C**, **Table S3**). Osteoclasts and fibroblasts identified previously barely express *C1Q* (**Figure 3B**). Then, we call these two groups of cells *C1Q*+ fibroblasts and *C1Q*+ osteoclasts since they express both *C1Q* and individual markers. They can also be special kinds of macrophages. The unknown cells have inconspicuous markers with a low fold change and uncertain gene ontology biological process (**Tables S3, 4**). The *FABP4*+ macrophages came from all 11 patients, a very small amount of which came from BC17 (**Figure 4D**). **Figure 3D** shows that the *FABP4*+ macrophage group mainly came from BC17. We consider that the two *FABP4*+ macrophage groups identified in twice clustering are the same. We compared marker genes of these cell groups and 34 DEGs obtained previously. There are 15 DEGs including *C1QA* found to be *C1Q*+ TAM markers **Figure 4E**, **Table S3**, 6 DEGs found to be monocyte markers. The GO enrichment of *C1Q*+ macrophage markers showed antigen processing and presentation and neutrophil activation (**Figure 4F**). We also set other cell groups to the GO analysis (**Table S4**).

### 3.4 *C1Q*+ TAM markers were better prognosis related and highly co-expressed

**Figure 5:**
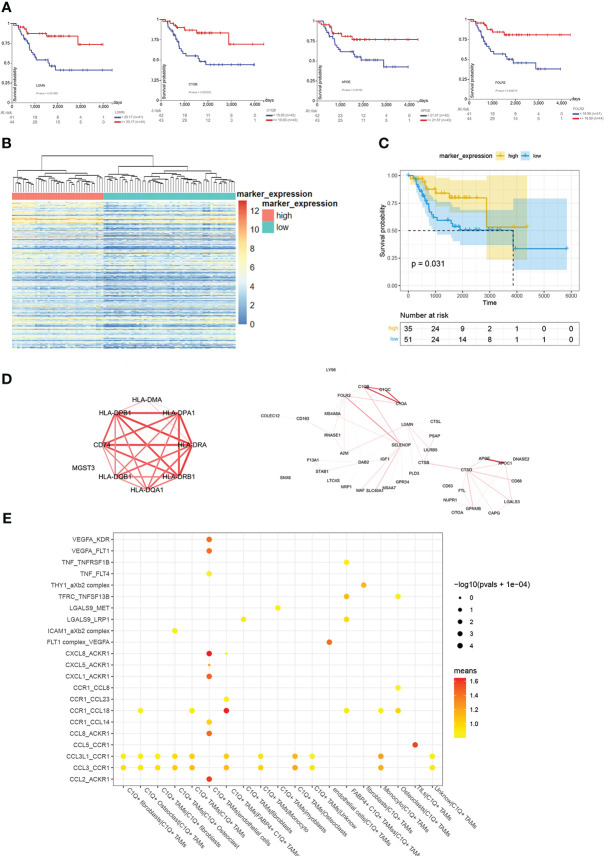
Overview of analyzing C1Q+ TAMs group and its marker. **(A)** The survival plots of *C1QA*, *C1QB*, *FOLR2*, *LGMN*, and *APOE*. **(B)** Cluster 88 examples in TARGET-OS into two groups by *C1Q*+ TAM markers with a fold change larger than 0.25. **(C)** The survival plot of high- and low- *C1Q*+ TAM infiltration groups. **(D)** The co-expression among *C1Q*+ TAM markers. The larger co-expression coefficient gets redder and wider lines. **(E)** Bubble plots show ligand–receptor pairs between *C1Q*+ TAMs and other cell groups.

*C1Q+* TAMs have 219 markers, we found that 5 of 10 top markers with the highest fold change were related to a better prognosis. They are *C1QA*, *C1QB*, *FOLR2*, *LGMN*, and *APOE* (**Figure 5A**). Similarly, we divided examples of dataset TARGET-OS into two groups by the result of the hierarchical cluster using the highly expressed markers of *C1Q*+ TAMs (**Figure 5B**). Moreover, the group with high *C1Q*+ macrophage marker expression showed a better overall survival (**Figure 5C**), which indicated that *C1Q*+ TAM infiltration plays an antitumor role. We calculated the co-expression coefficients between the 219 markers. We select *C1QA*, *C1QB*, and *C1QC* as a benchmark. The correlation coefficients among the three were 0.97, 0.97, and 0.98 in dataset TARGET-OS. In *C1Q*+ TAMs, it was 0.44. However, when we separated these *C1Q*+ TAMs by patients, the correlation coefficients of *C1Q*+ TAM markers from each patient evenly vary between 0.5 and 0.8 except BC2, BC3, and BC5 (**Table S5**). The difference could come from the heterogeneity of different patients’ TAMs and also the gene co-expressed between *C1Q*+ TAMs and other cells since other myeloid cells also express *C1QA/B/C*. We calculated the coefficients of the TAM markers for each patient, picked up the obvious co-expression genes, and drew the mean coefficients by Cytoscape (**Figure 5D**, **Table S6**). *CD74*, *HLA-D*, complement1, and apolipoprotein took the dominant role. Furthermore, we explored the cell–cell interactions and the ligand–receptor pairs between *C1Q*+ TAMs and other cell groups gained from the first clustering and subclustering (**Figure 5E**). Cell–cell interaction analysis by cellphone showed that *C1Q*+ TAMs mostly acted on endothelial cells. The ligand–receptor pairs are *CCL2/CCL8-ACKR1*, *CXCL1/5/8-ACKR1*, *VEGFA-KDR/FLT1*, *TNF-FLT4*, and *CCR1–CCL14.* We noticed that *C1Q*+ TAMs express higher *CCL2* and lower *VEGFA* and *CXCL8* (**Table S3**).

## 4 Discussion

The authors of dataset GSE21257 found that TAMs were associated with reduced metastasis and longer survival in high-grade osteosarcoma (33). We had similar findings, combining bulk datasets with a single-cell dataset. We reported more details about these TAMs in OS. Unlike many other kinds of tumors (15), we found that higher expressed *C1Q* was related to a better prognosis and the *C1Q*+ TAMs in OS were identified as an antitumor factor.

In the analysis of bulk datasets GSE21275 and GSE32981, we divided the examples into high- and low- immune infiltrated groups according to their hierarchical cluster results. Although the high-immune infiltrated groups showed that no kind of immune cell was lower infiltrated compared to the low-immune infiltrated groups, it showed better overall survival. Moreover, most of the DEGs were related to a better prognosis. These results portrayed an antitumor image of immune infiltration in OS. Next, we tried to identify immune components that play the most important role in the OS TME.

In order to minimize the error caused by the analysis method, we performed the differential expressing twice more using R packages edgeR and DESeq2 (**Figure S3**). We also mapped these DEGs in GSE152048 (**Figure S4**). They were mostly expressed by myeloid cells and C1Q+ TAMs. Then, we thought that the difference of the immune microenvironment is mainly caused by C1Q+ TAMs.

The analysis of GSE152048 showed that TAMs were the main immune cell population in OS. Further research showed that the DEGs gained from bulk datasets were enriched in *C1Q*+ TAMs, the markers of *C1Q*+ TAMs were related to a better prognosis, and the infiltration of *C1Q*+ TAMs went with better overall survival. These results indicated strongly that *C1Q*+ TAMs were just the main immune cell population against OS.

Although *C1Q* is regarded as a cancer-promoting factor (20), it has multiple regulatory effects on the immune system including inflammation and repair progress (18). *C1Q* could function as a pattern recognition receptor to opsonize apoptotic cells and extracellular vesicles. The extracellular vesicle-combined *C1Q* induces *IL-10* and *TGF-β* production in macrophages (24). Moreover, *IL-10* and *TGF-β* are known as immunosuppressive mediators and tumor promoters (34, 35). High-mobility group box 1 (*HMGB1*) and *HMGB1* plus *C1Q* can respectively regulate inflammatory macrophage polarization. *HMGB1* plus *C1Q* induced an anti-inflammatory phenotype by inhibiting *IRF5*, a regulator of pro-inflammatory macrophage polarization (36), when *HMGB1* singly induced a pro-inflammatory phenotype by upregulating IRF5 (37). As the trigger of the classical pathway of complement, C1 can produce C3a and C5a through cascade reaction. C3a and C5a can modulate the immune microenvironment toward a pro-tumor or antitumor response. Tumor type and local concentrations of the anaphylatoxins matter in this regulation (38).

Some research depicted the possible ways that *C1Q*+ TAMs promote or limit tumors. In colorectal cancer, the RNA N+6-methyladenosine (m6A) program can regulate *C1Q*+ TAMs, which express multiple immunomodulatory ligands to modulate tumor-infiltrating *CD8+* T cells. A low *METTL14-m+6A* level induces high levels of *EBI3*, a cytokine subunit, and finally leads to dysfunctional T cells (25). In clear-cell renal cell carcinoma, high densities of *C1Q*-producing TAMs contributed to the immunosuppressed microenvironment, in which a high expression of immune checkpoints was detected (39).

TAMs with different phenotypes could exert conversely on OS. M1 induced by interferon γ could secrete *HSPA1L* to promote OS cell apoptosis *via IRAK1* and *IRAK4 in vitro*. *HSPA1L* can be upregulated by *LGALS3BP* secreted by OS cells binding to *LGALS3* on M1 (40). M1 was thought to produce iNOS, oxygen intermediates, colony-stimulating factors, tumor necrosis factors, and interleukins to promote inflammation and to suppress OS. However, specific blockage of cytokines, nitric oxide, or reactive oxygen species did not inhibit the antitumor effect (41). M1 markers were found to enrich at the tumor interface region, whereas M2 markers were found to present throughout the whole tumor in OS pulmonary metastases (7). M2 could be recruited by *IL34* and promote osteosarcoma growth (42). *IL10*-polarized M2 could suppress OS in the presence of the anti-EGFR cetuximab (41). M2 enhanced metastasis of OS cells to the lungs in mice, and all-trans retinoic acid inhibited this metastasis *via* inhibiting the M2 polarization (43). *GNG12* was a highly effective biomarker for osteosarcoma; high *GNG12* related to a better prognosis and lower M1 and M2 scores (44). the Rab22a-NeoF1 fusion protein promotes M2 polarization by activating *STAT3* and subsequently facilitates lung metastases (45). In lung metastases, M2 correlated with curtailed patient survival could be induced by exosomes (4). Systemic administration of PLX3397, a *CSF1R* inhibitor, significantly suppressed the primary tumor growth and lung metastasis. After treatment, both M1 and M2 were depleted and the infiltration of *CD8*+T cells increased (46). Especially, *CD163*+ TAMs were reported to be crucial better prognostic biomarkers in OS (47). *CD163* was also a marker of *C1Q*+ TAMs (**Table S3**). We tried to find if there was a certain subtype of TAMs such as M1 and M2 among the *C1Q*+ TAMs as previous research summarized several conditions of macrophages and their markers (48–50). The difference in the direction of polarization macrophages has long been found, but we could not identify subclusters from *C1Q*+ TAMs; the markers of M1 and M2 did not show an obvious difference among them (**Figure S5**). Combined with the findings in bulk data, the samples were grouped according to immune-related genes, and then the immune cells of the two groups were scored. There were no immune cells with a high score in the low-immune infiltrated group. These genes include antitumor and pro-tumor genes, and cells also include antitumor and pro-tumor cells. If there were enough tumor-suppressor immune cells except TAMs in OS, the low-immune infiltrated group should have at least one main immune cell with high score. Bulk data suggest that immunosuppressive cells in OS are not easy to be observed. In the single-cell dataset, the classical M2 macrophages and M1 macrophages could not be clearly distinguished. There might be only one major immune cell in OS. They are *C1Q*+ TAMs, which suppress tumors. We noticed that some other researchers had worked on the TAM population in human breast cancer, which was defined by *APOE*, *APOC*, and *C1Q* expression. They found that a subset of *FOLR2*+ TAMs correlates with increased survival in patients with breast cancer. The *C1Q*+ TAMs and *FLOR2*+ TAMs described by our research and Nalio Ramos et al. are very similar. Both of them are defined by markers such as *APOE*, *FLOR2*, *CCL18*, *F13A1*, *MRC1*, *SLC40A1*, and *SELENOP* (*SEPP1*) (17). They described *FOLR2*+ TAMs as tissue-resident macrophages, whereas they failed to recognize M1- or M2-polarized macrophages in their dataset.

We tried to explain the tumor-limiting function of C1Q+TAMs by cell–cell interaction. Cellphone analysis suggested that C1Q+ TAMs act mainly on endothelial cells by the ACKR1-related pathway (**Figure 5E**). ACKR1 or DARC is a receptor for chemokines on erythrocytes and endothelial cells. It is not clear how ACKR1 expression contributes to the development and outcome of human diseases. At first, *ACKR1* was regarded as a neutralizer of chemokine instead of a signal transmitter, but now it is reported that chemokines retain their biological activity after binding to *DARC* [spice] (51, 52). When overexpressed in endothelial cells, *ACKR1* decreased the pro-angiogenic properties of chemokines (53). Further research about the interaction between TAMs and endothelial cells is required.

Some other subclusters of myeloid cells are also worth paying attention. We detected *FABP4*+ macrophages just like previous authors did (54). They have a small amount of 208 cells. The mean number of detected genes of these *FABP4*+ macrophages roared over 3,000, whereas the mean number of the rest was lower than 2,000. We thought that these cells were homologous doublets. The subcluster monocytes highly expressed *S100A8* and *S100A9*. *S100A8* and *S100A9*, molecular markers promoting pre-metastatic niche formation, can cause an expansion of myeloid-derived suppressor cells, thereby contributing to an immunocompromise (55, 56). There were 1,072 myeloid cells identified as *C1Q*+ osteoclasts, whereas there were 7,307 normal osteoclasts. Osteoclasts are multinucleated members of the monocyte/macrophage family, working as skeletal remodelers. OS cells mediated bone destruction by activated osteoclasts and obtained higher OS aggressiveness (57). However, in advanced OS, osteoclasts were proved to prevent metastatic osteosarcomas (58). Osteoclasts can secrete *C1Q* just like Kupffer cells in the liver and microglia in the brain. In turn, *C1Q* strongly promotes osteoclasts derived from monocytes (59). We noticed that there were *CD74*+*LUM*+*C1Q*+ cells. It might be a distinct TAM-induced extracellular matrix molecular signature (19). In the orthotopic colorectal cancer model, monocyte-derived TAMs promote tumor development by remodeling its extracellular matrix composition and structure (19).

## 5 Conclusion

This analysis revealed that a higher immune infiltration level improves the overall survival of OS patients and most of the high expression of immune infiltration-related genes links to better survival. Especially, we report *C1Q* as an antitumor factor in osteosarcoma. *C1Q*+ TAMs, marked by high *C1QA/B/C*, *APOE/C*, *FLOR2*, *SLC40A1*, *SEPP1*, and *MRC1* expression, contribute to a better prognosis in OS patients. *C1Q*+ TAMs are the major immune cells in the OS TME. This study provided the image of how immune cells influence prognosis in osteosarcoma and *C1Q*+ TAMs that can be therapeutic target cells to improve the osteosarcoma treatment.

## Data availability statement

Publicly available datasets were analyzed in this study. This data can be found here: TARGET-OS: https://portal.gdc.cancer.gov/repository GSE21257:https://www.ncbi.nlm.nih.gov/geo/query/acc.cgi?acc=GSE21257 GSE32981:https://www.ncbi.nlm.nih.gov/geo/query/acc.cgi?acc=GSE32981 GSE152048:https://www.ncbi.nlm.nih.gov/geo/query/acc.cgi?acc=GSE152048.

## Ethics statement

Ethical review and approval was not required for the study on human participants in accordance with the local legislation and institutional requirements. Written informed consent for participation was not required for this study in accordance with the national legislation and the institutional requirements.

## Author contributions

BL, JT, DW, and XZ designed this research. JT collected and analyzed datasets. DW drafted this manuscript. XZ adapted the manuscript for final submission. All authors contributed to the article and approved the submitted version.

## References

[1] Zheng D Yang K Chen X Li Y Chen Y. Analysis of immune-stromal score-based gene signature and molecular subtypes in osteosarcoma: Implications for prognosis and tumor immune microenvironment. Front Genet (2021) 12:699385. doi: 10.3389/fgene.2021.699385 34630511 PMC8495166

[2] Isakoff MS Bielack SS Meltzer P Gorlick R. Osteosarcoma: Current treatment and a collaborative pathway to success. J Clin (2015) 33(27):3029–35. doi: 10.1200/JCO.2014.59.4895 PMC4979196 26304877

[3] Corre I Verrecchia F Crenn V Redini F Trichet V. The osteosarcoma microenvironment: A complex but targetable ecosystem. Cells (2020) 9(4). doi: 10.3390/cells9040976 PMC7226971 32326444

[4] Wolf-Dennen K Gordon N Kleinerman ES. Exosomal communication by metastatic osteosarcoma cells modulates alveolar macrophages to an M2 tumor-promoting phenotype and inhibits tumoricidal functions. Oncoimmunology (2020) 9(1):1747677. doi: 10.1080/2162402X.2020.1747677 32313728 PMC7153823

[5] Majzner RG Simon JS Grosso JF Martinez D Pawel BR Santi M. Assessment of programmed death-ligand 1 expression and tumor-associated immune cells in pediatric cancer tissues. Cancer (2017) 123(19):3807–15. doi: 10.1002/cncr.30724 28608950

[6] Koirala P Roth ME Gill J Piperdi S Chinai JM Geller DS. Immune infiltration and PD-L1 expression in the tumor microenvironment are prognostic in osteosarcoma. Sci Rep (2016) 6(1):30093. doi: 10.1038/srep30093 27456063 PMC4960483

[7] Ligon JA Choi W Cojocaru G Fu W Hsiue EH-C Oke TF. Pathways of immune exclusion in metastatic osteosarcoma are associated with inferior patient outcomes. J Immunother Cancer (2021) 9(5):e001772. doi: 10.1136/jitc-2020-001772 34021032 PMC8144029

[8] Chen C Xie L Ren T Huang Y Xu J Guo W. Immunotherapy for osteosarcoma.: Fundamental mechanism, rationale, and recent breakthroughs. Cancer Lett (2021) 500:1–10. doi: 10.1016/j.canlet.2020.12.024 33359211

[9] Wang N Wang S Wang X Zheng Y Yang B Zhang J. Research trends in pharmacological modulation of tumor-associated macrophages. Clin Transl Med (2021) 11(1):e288–8. doi: 10.1002/ctm2.288 PMC7805405 33463063

[10] Xie R Ruan S Liu J Qin L Yang C Tong F. Furin-instructed aggregated gold nanoparticles for re-educating tumor associated macrophages and overcoming breast cancer chemoresistance. Biomaterials (2021) 275:120891. doi: 10.1016/j.biomaterials.2021.120891 34051669

[11] Figueiredo P Lepland A Scodeller P Fontana F Torrieri G Tiboni M. Peptide-guided resiquimod-loaded lignin nanoparticles convert tumor-associated macrophages from M2 to M1 phenotype for enhanced chemotherapy. Acta biomaterialia (2021) 133:231–43. doi: 10.1016/j.actbio.2020.09.038 33011297

[12] Cai Z Lim D Liu G Chen C Jin L Duan W. Valproic acid-like compounds enhance and prolong the radiotherapy effect on breast cancer by activating and maintaining anti-tumor immune function. Front Immunol (2021) 12:646384–4. doi: 10.3389/fimmu.2021.646384 PMC8149798 34054811

[13] Zhu Y Herndon JM Sojka DK Kim K-W Knolhoff BL Zuo C. Tissue-resident macrophages in pancreatic ductal adenocarcinoma originate from embryonic hematopoiesis and promote tumor progression. Immunity (2017) 47(2):323–338.e326. doi: 10.1016/j.immuni.2017.07.014 28813661 PMC5578409

[14] Bugatti M Bergamini M Missale F Monti M Laura A Pezzali I. A population of TIM4+FOLR2+ macrophages localized in tertiary lymphoid structures correlates to an active immune infiltrate across several cancer types. Cancer Immunol Res (2022) 10(11):1340–53. doi: 10.1158/2326-6066.CIR-22-0271 36122412

[15] Revel M Sautès-Fridman C Fridman W-H Roumenina LT. C1q+ macrophages: Passengers or drivers of cancer progression. Trends Cancer (2022) 8(7):517–26. doi: 10.1016/j.trecan.2022.02.006 35288093

[16] Blériot C Chakarov S Ginhoux F. Determinants of resident tissue macrophage identity and function. Immunity (2020) 52(6):957–70. doi: 10.1016/j.immuni.2020.05.014 32553181

[17] Nalio Ramos R Missolo-Koussou Y Gerber-Ferder Y Bromley CP Bugatti M Núñez NG. Tissue-resident FOLR2+ macrophages associate with CD8+ t cell infiltration in human breast cancer. Cell (2022) 185(7):1189–1207.e1125. doi: 10.1016/j.cell.2022.02.021 35325594

[18] Baldwin WM III Valujskikh A Fairchild RL. C1q as a potential tolerogenic therapeutic in transplantation. Am J Transplant (2021) 21(11):3519–23. doi: 10.1111/ajt.16705 PMC8564585 34058061

[19] Afik R Zigmond E Vugman M Klepfish M Shimshoni E Pasmanik-Chor M. Tumor macrophages are pivotal constructors of tumor collagenous matrix. J Exp Med (2016) 213(11):2315–31. doi: 10.1084/jem.20151193 PMC5068227 27697834

[20] Bulla R Tripodo C Rami D Ling GS Agostinis C Guarnotta C. C1q acts in the tumour microenvironment as a cancer-promoting factor independently of complement activation. Nat Commun (2016) 7:10346. doi: 10.1038/ncomms10346 26831747 PMC4740357

[21] Markiewski MM DeAngelis RA Benencia F Ricklin-Lichtsteiner SK Koutoulaki A Gerard C. Modulation of the antitumor immune response by complement. Nat Immunol (2008) 9(11):1225–35. doi: 10.1038/ni.1655 PMC2678913 18820683

[22] Afshar-Kharghan V. The role of the complement system in cancer. J Clin Invest (2017) 127(3):780–9. doi: 10.1172/JCI90962 PMC5330758 28248200

[23] Reis ES Mastellos DC Ricklin D Mantovani A Lambris JD. Complement in cancer: Untangling an intricate relationship. Nat Rev Immunol (2018) 18(1):5–18. doi: 10.1038/nri.2017.97 28920587 PMC5816344

[24] Bohlson SS O’Conner SD Hulsebus HJ Ho M-M Fraser DA. Complement, C1q, and C1q-related molecules regulate macrophage polarization. Front Immunol (2014) 5. doi: 10.3389/fimmu.2014.00402 PMC4139736 25191325

[25] Dong L Chen C Zhang Y Guo P Wang Z Li J. The loss of RNA N6-adenosine methyltransferase Mettl14 in tumor-associated macrophages promotes CD8+ t cell dysfunction and tumor growth. Cancer Cell (2021) 39(7):945–957.e910. doi: 10.1016/j.ccell.2021.04.016 34019807

[26] Charoentong P Finotello F Angelova M Mayer C Efremova M Rieder D. Pan-cancer immunogenomic analyses reveal genotype-immunophenotype relationships and predictors of response to checkpoint blockade. Cell Rep (2017) 18(1):248–62. doi: 10.1016/j.celrep.2016.12.019 28052254

[27] Ritchie ME Phipson B Wu D Hu Y Law CW Shi W. Limma powers differential expression analyses for RNA-sequencing and microarray studies. Nucleic Acids Res (2015) 43(7):e47–7. doi: 10.1093/nar/gkv007 PMC4402510 25605792

[28] Love MI Huber W Anders S. Moderated estimation of fold change and dispersion for RNA-seq data with DESeq2. Genome Biol (2014) 15(12):550. doi: 10.1186/s13059-014-0550-8 25516281 PMC4302049

[29] Robinson MD McCarthy DJ Smyth GK. EdgeR: @ a bioconductor package for differential expression analysis of digital gene expression data. Bioinf (Oxford England) (2010) 26(1):139–40. doi: 10.1093/bioinformatics/btp616 PMC2796818 19910308

[30] Subramanian A Tamayo P Mootha VK Mukherjee S Ebert BL Gillette MA. Gene set enrichment analysis. a knowledge-based approach for interpreting genome-wide expression profiles. Proc Natl Acad Sci U.S.A. (2005) 102(43):15545–50. doi: 10.1073/pnas.0506580102 PMC1239896 16199517

[31] McGinnis CS Murrow LM Gartner ZJ. DoubletFinder.: Doublet detection in single-cell rna sequencing data using artificial nearest neighbors. Cell Syst (2019) 8(4):329–337.e324. doi: 10.1016/j.cels.2019.03.003 30954475 PMC6853612

[32] Vento-Tormo R Efremova M Botting RA Turco MY Vento-Tormo M Meyer KB. Single-cell reconstruction of the early maternal-fetal interface in humans. Nature (2018) 563(7731):347–53. doi: 10.1038/s41586-018-0698-6 PMC7612850 30429548

[33] Buddingh EP Kuijjer ML Duim RAJ Bürger H Agelopoulos K Myklebost O. Tumor-infiltrating macrophages are associated with metastasis suppression in high-grade osteosarcoma: A rationale for treatment with macrophage activating agents. Clin Cancer Res (2011) 17(8):2110–9. doi: 10.1158/1078-0432.CCR-10-2047 21372215

[34] Locati M Curtale G Mantovani A. Diversity, mechanisms, and significance of macrophage plasticity. Annu Rev Pathol (2020) 15:123–47. doi: 10.1146/annurev-pathmechdis-012418-012718 PMC7176483 31530089

[35] Batlle E Massagué J. Transforming growth factor-β signaling in immunity and cancer. Immunity (2019) 50(4):924–40. doi: 10.1016/j.immuni.2019.03.024 PMC7507121 30995507

[36] Hedl M Yan J Witt H Abraham C. IRF5 is required for bacterial clearance in human M1-polarized macrophages, and IRF5 immune-mediated disease risk variants modulate this outcome. J Immunol (2019) 202(3):920–30. doi: 10.4049/jimmunol.1800226 PMC6585431 30593537

[37] Liu T Xiang A Peng T Doran AC Tracey KJ Barnes BJ. HMGB1-C1q complexes regulate macrophage function by switching between leukotriene and specialized proresolving mediator biosynthesis. Proc Natl Acad Sci U.S.A. (2019) 116(46):23254–63. doi: 10.1073/pnas.1907490116 PMC6859319 31570601

[38] Roumenina LT Daugan MV Petitprez F Sautès-Fridman C Fridman WH. Context-dependent roles of complement in cancer. Nat Rev Cancer (2019) 19(12):698–715. doi: 10.1038/s41568-019-0210-0 31666715

[39] Roumenina LT Daugan MV Noé R Petitprez F Vano YA Sanchez-Salas R. Tumor cells hijack macrophage-produced complement C1q to promote tumor growth. Cancer Immunol Res (2019) 7(7):1091–105. doi: 10.1158/2326-6066.CIR-18-0891 31164356

[40] Li J Zhao C Li Y Wen J Wang S Wang D. Osteosarcoma exocytosis of soluble LGALS3BP mediates macrophages toward a tumoricidal phenotype. Cancer Lett (2022) 528:1–15. doi: 10.1016/j.canlet.2021.12.023 34952143

[41] Pahl JHW Kwappenberg KMC Varypataki EM Santos SJ Kuijjer ML Mohamed S. Macrophages inhibit human osteosarcoma cell growth after activation with the bacterial cell wall derivative liposomal muramyl tripeptide in combination with interferon-γ. J Exp Clin Cancer Res (2014) 33(1):27–7. doi: 10.1186/1756-9966-33-27 PMC4007518 24612598

[42] Ségaliny AI Mohamadi A Dizier B Lokajczyk A Brion R Lanel R. Interleukin-34 promotes tumor progression and metastatic process in osteosarcoma through induction of angiogenesis and macrophage recruitment. Int J Cancer (2015) 137(1):73–85. doi: 10.1002/ijc.29376 25471534

[43] Zhou Q Xian M Xiang S Xiang D Shao X Wang J. All-trans retinoic acid prevents osteosarcoma metastasis by inhibiting m2 polarization of tumor-associated macrophages. Cancer Immunol Res (2017) 5(7):547–59. doi: 10.1158/2326-6066.CIR-16-0259 28515123

[44] Yuan J Yuan Z Ye A Wu T Jia J Guo J. Low GNG12 expression predicts adverse outcomes: A potential therapeutic target for osteosarcoma. Front Immunol (2021) 12:758845–5. doi: 10.3389/fimmu.2021.758845 PMC8527884 34691083

[45] Zhong L Liao D Li J Liu W Wang J Zeng C. Rab22a-NeoF1 fusion protein promotes osteosarcoma lung metastasis through its secretion into exosomes. Signal Transduct Target Ther (2021) 6(1):59–9. doi: 10.1038/s41392-020-00414-1 PMC7876000 33568623

[46] Fujiwara T Yakoub MA Chandler A Christ AB Yang G Ouerfelli O. CSF1/CSF1R signaling inhibitor pexidartinib (PLX3397) reprograms tumor-associated macrophages and stimulates t-cell infiltration in the sarcoma microenvironment. Mol Cancer Ther (2021) 20(8):1388–99. doi: 10.1158/1535-7163.MCT-20-0591 PMC9336538 34088832

[47] Gomez-Brouchet A Illac C Gilhodes J Bouvier C Aubert S Guinebretiere JM. CD163-positive tumor-associated macrophages and CD8-positive cytotoxic lymphocytes are powerful diagnostic markers for the therapeutic stratification of osteosarcoma patients: An immunohistochemical analysis of the biopsies fromthe french OS2006 phase 3 trial. Oncoimmunology (2017) 6(9):e1331193. doi: 10.1080/2162402X.2017.1331193 28932633 PMC5599091

[48] Wang C Ma C Gong L Guo Y Fu K Zhang Y. Macrophage polarization and its role in liver disease. Front Immunol (2021) 12:803037–7. doi: 10.3389/fimmu.2021.803037 PMC8712501 34970275

[49] Mohapatra S Pioppini C Ozpolat B Calin GA. Non-coding RNAs regulation of macrophage polarization in cancer. Mol Cancer (2021) 20(1):24–4. doi: 10.1186/s12943-021-01313-x PMC7849140 33522932

[50] Zhou K Cheng T Zhan J Peng X Zhang Y Wen J. Targeting tumor-associated macrophages in the tumor microenvironment. Oncol Lett (2020) 20(5):234–4. doi: 10.3892/ol.2020.12097 PMC7500051 32968456

[51] Rot A. Contribution of duffy antigen to chemokine function. Cytokine Growth Factor Rev (2005) 16(6):687–94. doi: 10.1016/j.cytogfr.2005.05.011 16054417

[52] Groblewska M Litman-Zawadzka A Mroczko B. The role of selected chemokines and their receptors in the development of gliomas. Int J Mol Sci (2020) 21(10). doi: 10.3390/ijms21103704 PMC7279280 32456359

[53] Du J Luan J Liu H Daniel TO Peiper S Chen TS. Potential role for duffy antigen chemokine-binding protein in angiogenesis and maintenance of homeostasis in response to stress. J leukocyte Biol (2002) 71(1):141–53. doi: 10.1189/jlb.71.1.141 PMC2665273 11781390

[54] Zhou Y Yang D Yang Q Lv X Huang W Zhou Z. Single-cell RNA landscape of intratumoral heterogeneity and immunosuppressive microenvironment in advanced osteosarcoma. Nat Commun (2020) 11(1):6322–2. doi: 10.1038/s41467-020-20059-6 PMC7730477 33303760

[55] Heinemann AS Pirr S Fehlhaber B Mellinger L Burgmann J Busse M. In neonates S100A8/S100A9 alarmins prevent the expansion of a specific inflammatory monocyte population promoting septic shock. FASEB J (2017) 31(3):1153–64. doi: 10.1096/fj.201601083R 27993995

[56] Zhao F Hoechst B Duffy A Gamrekelashvili J Fioravanti S Manns MP. S100A9 a new marker for monocytic human myeloid-derived suppressor cells. Immunology (2012) 136(2):176–83. doi: 10.1111/j.1365-2567.2012.03566.x PMC3403264 22304731

[57] Alfranca A Martinez-Cruzado L Tornin J Abarrategi A Amaral T de Alava E. Bone microenvironment signals in osteosarcoma development. Cell Mol Life Sci (2015) 72(16):3097–113. doi: 10.1007/s00018-015-1918-y PMC11113487 25935149

[58] Endo-Munoz L Cumming A Rickwood D Wilson D Cueva C Ng C. Loss of osteoclasts contributes to development of osteosarcoma pulmonary metastases. Cancer Res (2010) 70(18):7063–72. doi: 10.1158/0008-5472.CAN-09-4291 20823153

[59] Teo BHD Bobryshev Yuri V Teh Boon K Wong Siew H Lu J. Complement C1q production by osteoclasts and its regulation of osteoclast development. Biochem J (2012) 447(2):229–37. doi: 10.1042/BJ20120888 22812635

