# Supplementary figures and images for: Single-cell RNA datasets and bulk RNA datasets analysis demonstrated C1Q+ tumor-associated macrophage as a major and antitumor immune cell population in osteosarcoma
Source: Front Immunol. 2023 Feb 6;14:911368. doi: 10.3389/fimmu.2023.911368 (PMC9939514; doi:10.3389/fimmu.2023.911368)

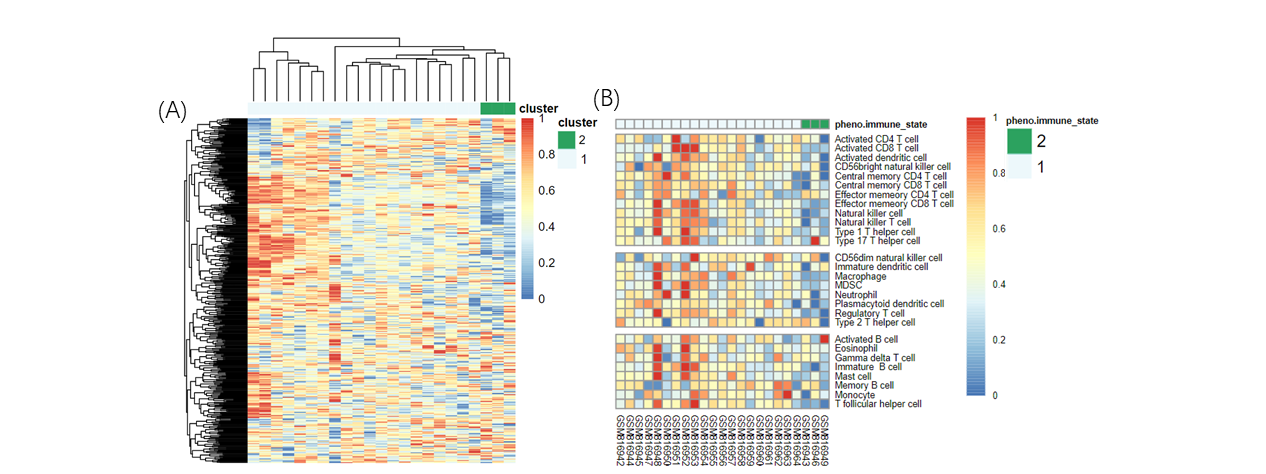

Supplement: Supplementary file 1 [file Image_1.tif]

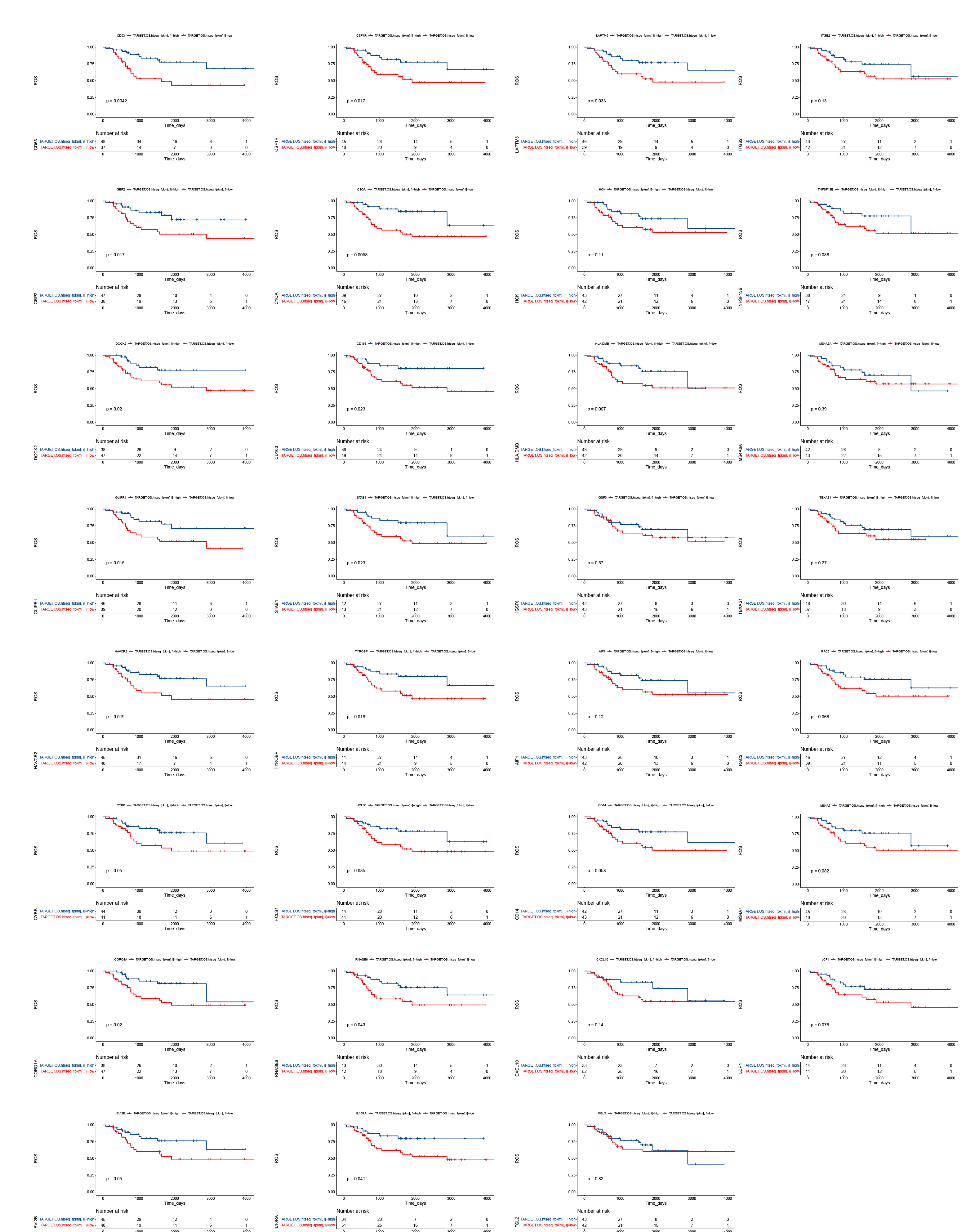

Supplement: Supplementary file 2 [file Image_2.tif]

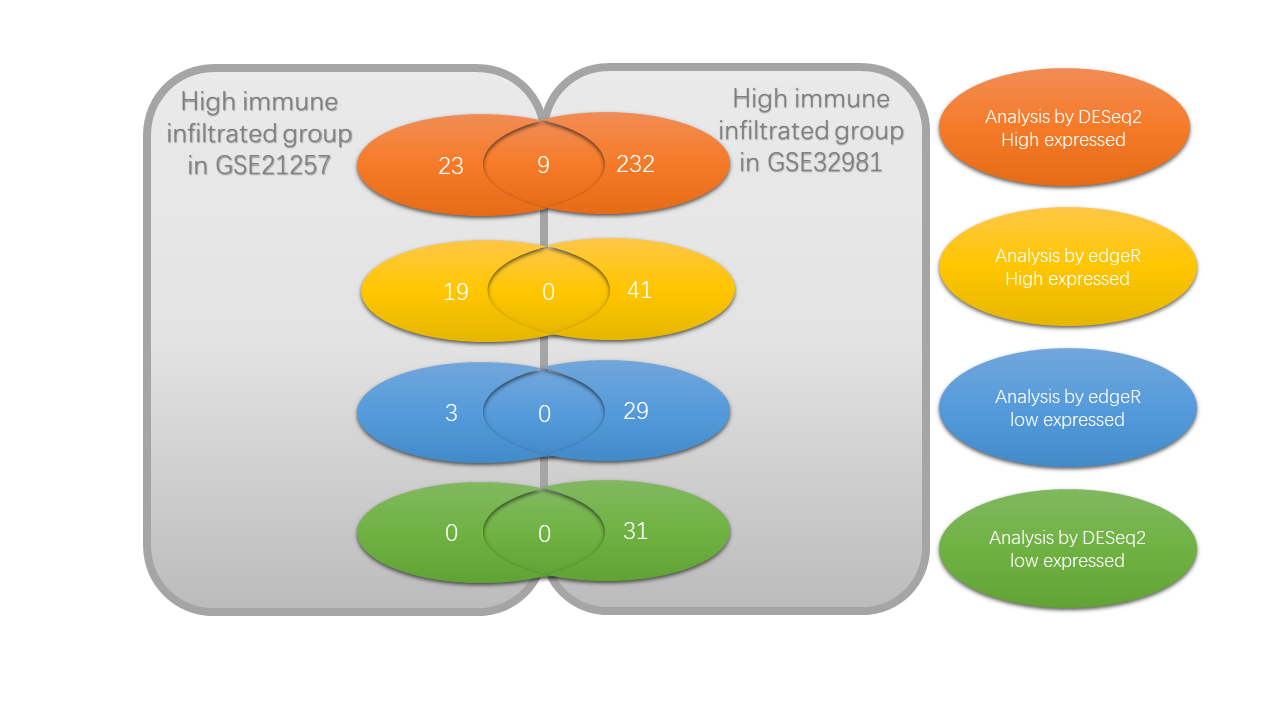

Supplement: Supplementary file 3 [file Image_3.tif]

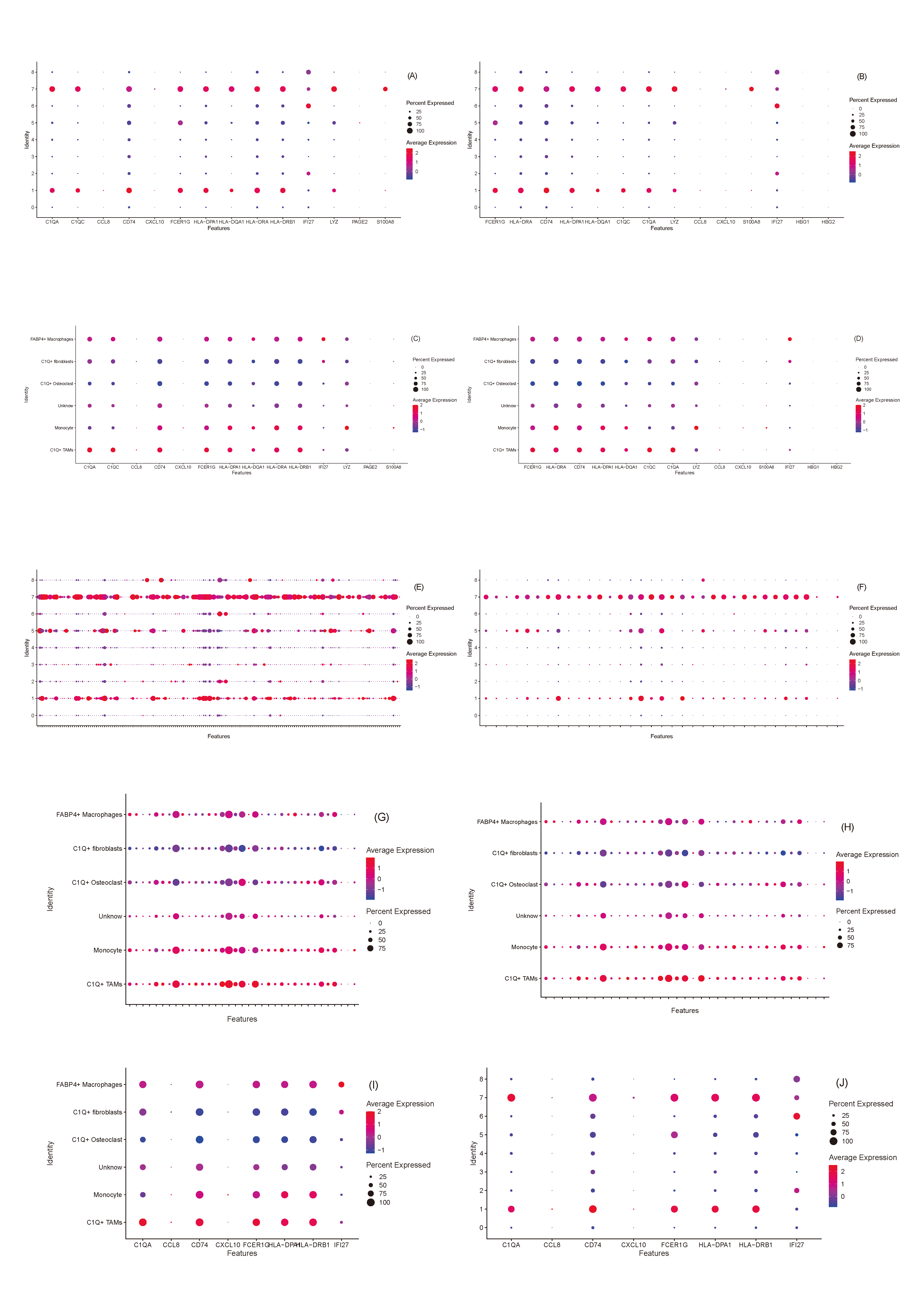

Supplement: Supplementary file 4 [file Image_4.tif]

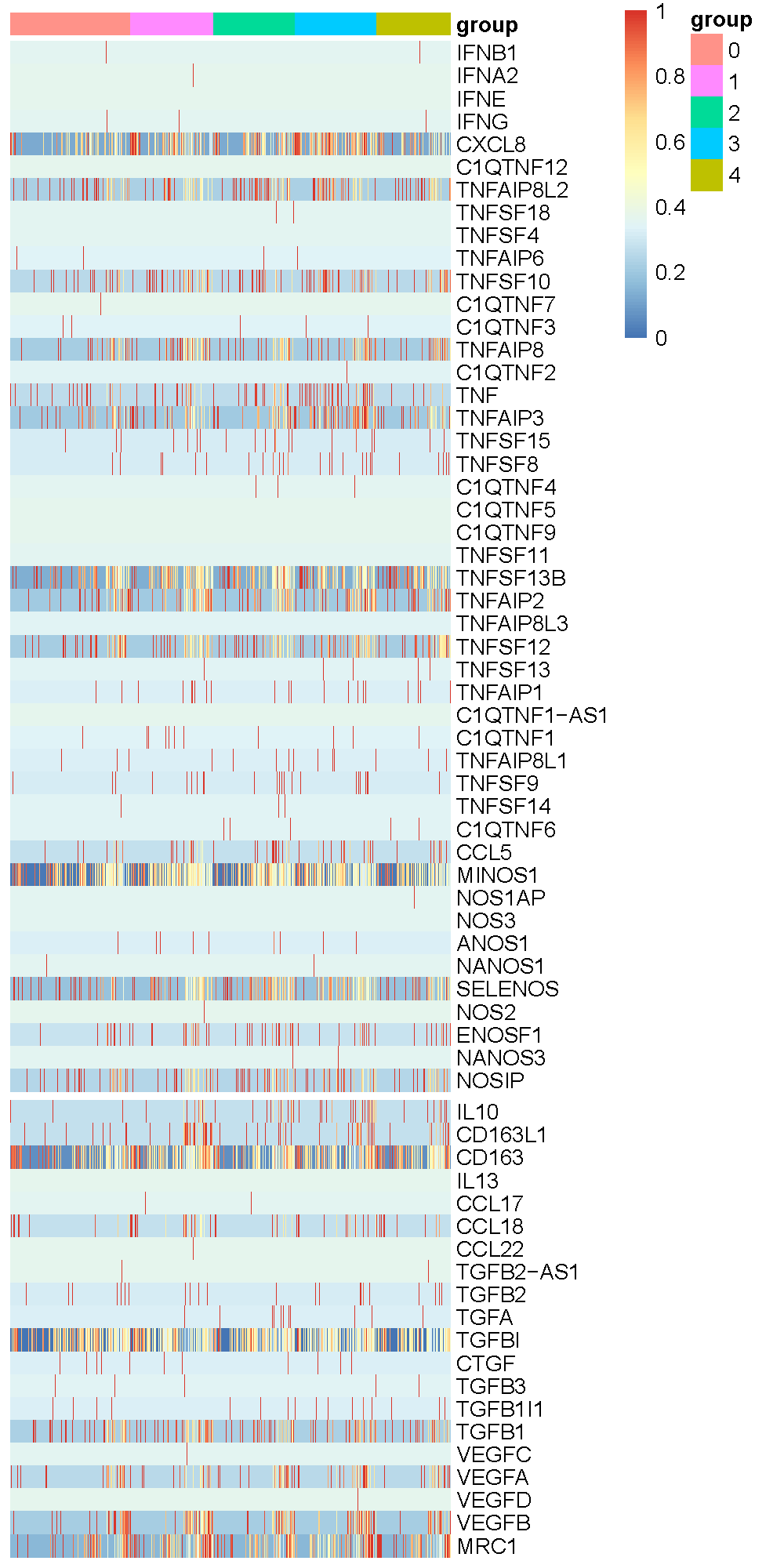

Supplement: Supplementary file 5 [file Image_5.tif]
