# Supplementary material for: Single-cell RNA datasets and bulk RNA datasets analysis demonstrated C1Q+ tumor-associated macrophage as a major and antitumor immune cell population in osteosarcoma
Source: Front Immunol. 2023 Feb 6;14:911368. doi: 10.3389/fimmu.2023.911368 (PMC9939514; doi:10.3389/fimmu.2023.911368)
Supplement: Supplementary file 14 [file DataSheet_1.docx]

Supplementary Material

# Supplementary Data

The R code and all R data was packed together. R code was annotated according to Figures and tables. DOI: 10.6084/m9.figshare.19498865

# Supplementary Figures and Tables

## Supplementary Figures

**Supplementary Figure 1:** Figure S1 (A). Cluster examples in GSE32981 into two groups by immune metagenes. (B). The score of 28 kinds of immune cells calculated by ssGSEA

**Supplementary Figure 2:** survival plots of overlapped DEGs relates to prognosis

**Supplementary Figure 3:** besides R package limma, the differential expressing analyses were also performed on R package edgeR and DESeq2. We performed the same analyses on both GSE21257 and GSE32981. By DESeq2, we found that high immune infiltrated group in GSE21257/GSE32981 highly expressed 23/232 genes and expressed lower 0/31 genes, which were represented by orange and blue ovals. The yellow and blue oval represent the condition using edgeR. Using limma, we found 34 overlapped DEGs. For comparison, we found 9 overlapped DEGs using DESeq2 and 0 using edgeR.

**Supplementary Figure 4:** Figure S4 Dot plots of differential expressed genes between high and low immune infiltrated examples. We found that DEGs were mostly expressed by myeloid and C1Q+ TAMs(A). Dot plot of the DEGs showed their expressing proportion and level among 9 clusters ((0-OS, 1-myeloid, 2-fibroblasts, 3-TILs including T and NK cells, 4-proliferating OS, 5-osteoclasts, 6-endothelial cells, 7-FABP4+ macrophages, 8-myoblasts). R package: edgeR. Dataset: GSE21257.

Figure S4 (B). Dot plot of the DEGs showed their expressing proportion and level among 9 clusters ((0-OS, 1-myeloid, 2-fibroblasts, 3-TILs including T and NK cells, 4-proliferating OS, 5-osteoclasts, 6-endothelial cells, 7-FABP4+ macrophages, 8-myoblasts). R package: DESeq2. Dataset: GSE21257.

Figure S4 (C). Dot plot of the DEGs showed their expressing proportion and level among 6 clusters. R package: edgeR. Dataset: GSE21257.

Figure S4 (D). Dot plot of the DEGs showed their expressing proportion and level among 6 clusters. R package: DESeq2. Dataset: GSE21257.

Figure S4 (E). Dot plot of the DEGs showed their expressing proportion and level among 9 clusters ((0-OS, 1-myeloid, 2-fibroblasts, 3-TILs including T and NK cells, 4-proliferating OS, 5-osteoclasts, 6-endothelial cells, 7-FABP4+ macrophages, 8-myoblasts). R package: DESeq2. Dataset: GSE32981.

Figure S4 (F). Dot plot of the DEGs showed their expressing proportion and level among 9 clusters ((0-OS, 1-myeloid, 2-fibroblasts, 3-TILs including T and NK cells , 4-proliferating OS, 5-osteoclasts, 6-endothelial cells, 7-FABP4+ macrophages, 8-myoblasts). R package: edgeR. Dataset: GSE32981.

Figure S4 (G). Dot plot of the DEGs showed their expressing proportion and level among 6 clusters. R package: DESeq2. Dataset: GSE32981.

Figure S4 (H). Dot plot of the DEGs showed their expressing proportion and level among 6 clusters. R package: edgeR. Dataset: GSE32981.

Figure S4 (I). Dot plot of the overlapped DEGs, which were highly expressed in high immune infiltrated groups of both GSE21257 and GSE32981, showed their expressing proportion and level among 9 clusters ((0-OS, 1-myeloid, 2-fibroblasts, 3-TILs including T and NK cells, 4-proliferating OS, 5-osteoclasts, 6-endothelial cells, 7-FABP4+ macrophages, 8-myoblasts). R package: DESeq2.

Figure S4 (J). Dot plot of the overlapped DEGs, which were highly expressed in high immune infiltrated groups of both GSE21257 and GSE32981, showed their expressing proportion and level among 6 clusters. R package: DESeq2.

**Supplementary Figure 5:** C1Q+ TAMs were clustered into 5 groups. These groups cannot be marked by M1 or M2 markers

# Supplementary Tables

**Supplementary Table 1:** all markers of all 9 clusters identified from 99668 cells

**Supplementary Table 2:** GO-BP results of the markers of 9 clusters identified from 99668 cells

**Supplementary Table 3:** all markers of 6 cell groups identified from myeloid cells

**Supplementary Table 4:** GO-BP results of the markers of 6 cell groups identified from myeloid cells

**Supplementary Table 5:** the correlation coefficient among *C1QA*, *C1QB*, *C1QC* in 11 OS patients

**Supplementary Table 6:** the mean correlation coefficient of *C1Q*+ TAM markers

**Supplementary Table 7:** clinical information of patients of dataset GSE21257

**Supplementary Table 8:** clinical information of patients of dataset TARGET-OS
